# The cost of vaccination throughout life: A western European overview

**DOI:** 10.1080/21645515.2016.1154649

**Published:** 2016-04-06

**Authors:** Olivier Ethgen, Murielle Cornier, Emilie Chriv, Florence Baron-Papillon

**Affiliations:** a Department of Public Health Sciences, Faculty of Medicine, University of Liège, Liège, Belgium, and SERFAN Innovation, Namur, Belgium; b Sanofi Pasteur MSD, Lyon, France; c Market Access Solutions, London, UK

**Keywords:** cost, healthcare perspective, national vaccination calendar, vaccination, Western Europe

## Abstract

Despite the interest of policy makers, the actual investment in vaccination is poorly documented. Our study assessed the costs of vaccination throughout life for a fully immunized Western European citizen. National vaccination calendars for England, France, Germany, Italy, Portugal, Spain and Sweden were retrieved. We differentiated men from women and healthy individuals from those suffering from underlying conditions who require specific additional vaccinations. Vaccine costs and administration fees were retrieved from official national source and calculated from the national healthcare perspective. Vaccinating an individual against up to 17 diseases throughout his entire life and in full compliance with national vaccination calendars cost between €328 and €2,352 (vaccines costs only) and between €443 and €3,395 (administration costs included), the lowest range corresponds to a healthy man in Sweden and the highest to a woman with underlying conditions in England. Vaccination costs varied among countries due to heterogeneous national vaccination calendars and organization. In all countries, adults (18–64 y) and elderly (≥65 y) accounted for the lowest vaccines costs compared with infants (0–24 m) and children/adolescents (2–17 y). In comparison, other mass secondary preventive therapies may be at least 3 times more costly. Vaccination requires a relatively low level of investment per individual. Our estimates should be considered to be the maximum potential costs due to our 100% compliance assumption. Increasing coverage rates would bring additional public health benefits for a relatively low incremental cost. A life-course approach of vaccination should also be encouraged because some missed opportunities remain in senior vaccinations.

## Introduction

Vaccination is among the mainstays of prevention and remains the best way to keep the population healthy and away from costly medical care.^1^ Currently, 25 communicable diseases are vaccine-preventable. Other diseases have even been eradicated through vaccination policies, such as wild-type poliovirus or *Haemophilus influenza* type B. Thus, vaccines have reduced the incidence of many infectious diseases and their associated mortality, morbidity and economic burden.^2^ As such, they significantly contribute to the sustainability of healthcare systems and to economic growth by maintaining a productive and healthier population.^3-6^

Like many developed countries, Western European countries have established vaccination calendars to protect their population against the threats of infectious diseases. The implementation of a vaccination calendar to protect people against approximately 20 infectious pathogens requires appropriate dedicated resources. Each year, resources for vaccine purchase, administration, and logistics are mobilized by healthcare professionals.^7^

Policy makers have been paying more and more attention to the cost of healthcare because of their shrinking budgetary capability.^8^ This is even more manifest since the economic crisis hit Europe in 2008.^9^ The actual investment in vaccinations as a whole, which is the cost encompassing the entire vaccine calendar in place in a specific country for its residents, has been rarely addressed in the literature and remains poorly documented even by policy makers.

Economic studies usually remain focused on a newly launched single vaccine or one that has been added to the vaccination calendar and tends to report and compare the average healthcare cost per individual.^10-13^ However, in our contemporary budget-conscious era, knowing the level of investment required to immunize individuals throughout their lifetime in accordance with vaccination calendars can be helpful to budget vaccination policies.^14^

The objective of this study was to estimate the individual lifetime cost of vaccination in Western Europe from a National Health Insurance (NHI) perspective. We took a theoretical approach in the sense that we considered the cost for an individual immunized in full compliance with national vaccination calendars.

## Results

The different national vaccination calendars (NVC) recommend the vaccination against 10 to 17 pathogens in total. The lowest number of pathogens vaccinated against was found in Sweden for healthy individuals, and the highest number was found in Germany for individuals with an underlying condition (Table 1).

**Table 1.** Diseases prevented according to national vaccination calendars (2014 or 2015).

	Germany		England		France		Italy		Spain		Sweden		Portugal	
	*Healthy*	*UC*	*Healthy*	*UC*	*Healthy*	*UC*	*Healthy*	*UC*	*Healthy*	*UC*	*Healthy*	*UC*	*Healthy*	*UC*
Diphtheria	✓	✓	✓	✓	✓	✓	✓	✓	✓	✓	✓	✓	✓	✓
Tetanus	✓	✓	✓	✓	✓	✓	✓	✓	✓	✓	✓	✓	✓	✓
Poliomyelitis	✓	✓	✓	✓	✓	✓	✓	✓	✓	✓	✓	✓	✓	✓
Pertussis	✓	✓	✓	✓	✓	✓	✓	✓	✓	✓	✓	✓	✓	✓
Hemophilius influenza B	✓	✓	✓	✓	✓	✓	✓	✓	✓	✓	✓	✓	✓	✓
Influenza	✓^1^	✓	✓^3^	✓	✓^5^	✓	✓^5^	✓	✓^5^	✓			✓^5^	✓
Pneumococcal	✓	✓	✓	✓	✓	✓	✓	✓	✓	✓	✓	✓	✓	✓
Meningococcal C	✓		✓	✓	✓	✓	✓	✓	✓	✓			✓	✓
Meningococcal ACWY		✓												
Measle	✓	✓	✓	✓	✓	✓	✓	✓	✓	✓	✓	✓	✓	✓
Mumps	✓	✓	✓	✓	✓	✓	✓	✓	✓	✓	✓	✓	✓	✓
Rubella	✓	✓	✓	✓	✓	✓	✓	✓	✓	✓	✓	✓	✓	✓
Varicella	✓	✓				✓		✓		✓				
Hepatitis A		✓				✓		✓						✓
Hepatitis B	✓	✓		✓	✓	✓	✓	✓		✓		✓	✓	✓
Rotavirus	✓	✓	✓	✓										
HPV^4^	✓	✓	✓	✓	✓	✓	✓	✓	✓	✓	✓	✓	✓	✓
Zoster			✓^2^	✓^2^										
Tuberculosis						✓						✓	✓	✓
Tick-borne encephalitis		✓												

UC: individuals with underlying conditions;

1 ≥60y only;

2 70y only;

3 2–4y & ≥65y only;

4 Girls only;

5 ≥65y only

**Figure 1.**
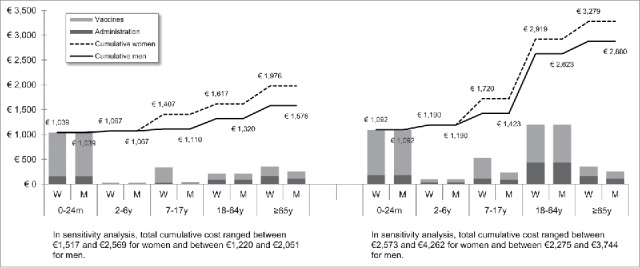
Maximal vaccination cost throughout life in Germany.

**Figure 2.**
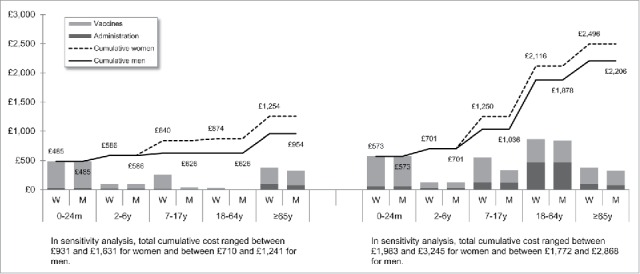
Maximal vaccination cost throughout life in England.

**Figure 3.**
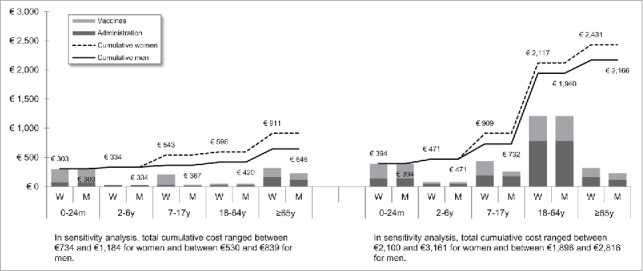
Maximal vaccination cost throughout life in France.

**Figure 4.**
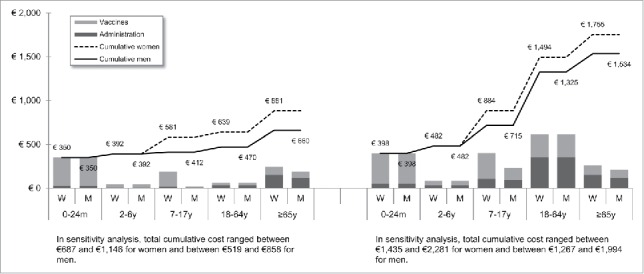
Maximal vaccination cost throughout life in Italy.

**Figure 5.**
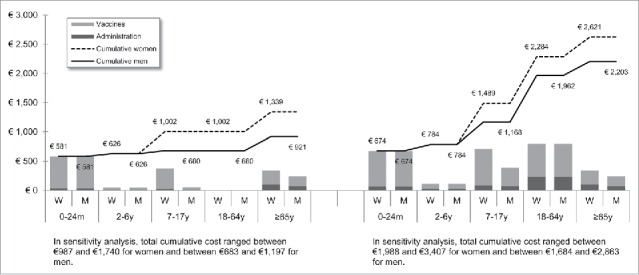
Maximal vaccination cost throughout life in Spain.

**Figure 6.**
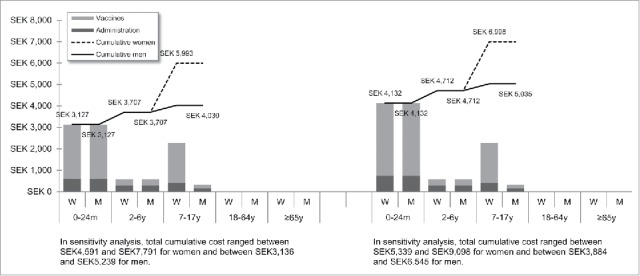
Maximal vaccination cost throughout life in Sweden.

**Figure 7.**
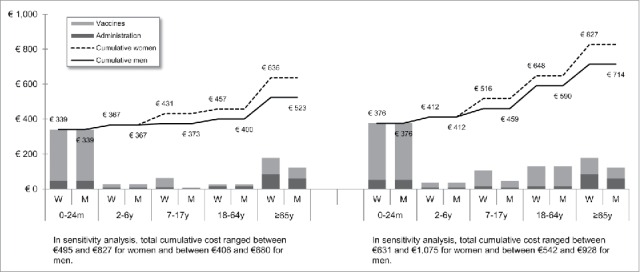
Maximal vaccination cost throughout life in Portugal.

Figures 1 to 7 show the estimated theoretical cost of vaccination throughout life (vaccine acquisition and administration) per gender and per country. Vaccinating an individual throughout her/his entire lifespan and in full compliance with NVC would range from €443 to vaccinate against 10 pathogens in healthy men in Sweden (with SEK1 = €0.11) to €3,395 to vaccinate against 15 pathogens in women with underlying conditions in England (with £1 = €1.36). This represents a range of €44 to €226 per pathogen vaccinated against in the selected countries. In the sensitivity analyses, these costs ranged between €345 (€35/pathogen vaccinated against) and €576 (€58/pathogen vaccinated against) in Sweden and between €2,697 (€180/pathogen vaccinated against) and €4,413 (€294/pathogen vaccinated against) in England.

We split the cost of vaccination between vaccine procurement and administration cost. The vaccine purchase ranged from €328 (healthy men in Sweden) to €2,352 in Germany (women with underlying conditions). The administration costs ranged from €115 (healthy men in Sweden) to €1,328 in France (women with underlying conditions). Administration represented 12% to 40% of the entire vaccination cost in healthy individuals and 20% to 58% of the entire cost in individuals with underlying conditions. These lower and upper estimates were found for Spanish women and French men, respectively.

In France, a significant proportion of the vaccine cost is not fully borne by the NHI but by complementary insurance and/or individuals. Indeed, nearly 35% of the vaccine cost is paid by individual (except for the combined Measles-Mumps-Rubella vaccine in infants (0–24 m) and the flu vaccine in individuals with underlying conditions and in the elderly (≥65 y) that are covered at 100% by the NHI). In Portugal, parents of infants below 24 m and suffering from underlying conditions are required to cover 63% of the cost of the hepatitis A vaccine. The influenza vaccine is also only partially funded by the NHI (at 37%) for individuals under the age of 65 y and for individuals with underlying conditions. In all other countries, the NHIs cover up to 100% of the cost of vaccination under their respective vaccination calendars.

In healthy individuals, the highest costs were found in pediatric vaccinations compared with other age groups (between €303 in France and €1,039 in Germany for the 0–24 m age group). In individuals with underlying conditions, and for all countries but Sweden and Portugal, adults (18–64 y) had the highest vaccination costs because the influenza vaccination was recommended annually.

Across all countries and due to the HPV vaccination, women were consistently found to have higher cumulative vaccination costs than men from adolescence (7–17 y). This difference was slightly increased in the elderly (≥65 y) because of the higher life expectancy of women and the resulting higher number of influenza vaccinations.

## Discussion

To our knowledge, this study is among the first that has systematically documented the cost of vaccinations over the entire life span, and has considered established national vaccination calendars and not just a single vaccine. In addition to the health economic evaluation (under the form of a cost-effectiveness analysis), our findings could prove useful for policymakers in future financial planning and evaluation of nationwide vaccination calendars.

Vaccinating an individual over his/her lifetime and in full compliance with the recommended vaccine calendar would not exceed €443 (€44/disease prevented) for a healthy man in Sweden and €3,395 (€226/disease prevented) for a woman suffering from an underlying condition in England. Vaccination costs were variable among countries due to heterogeneous vaccination calendars and organization. These countries do not have the same prevention scope and policies. The number of pathogens vaccinated against by national vaccination recommendations range from 10 in Sweden for healthy individuals up to 17 in Germany for individuals suffering from one or more underlying conditions. In addition, the funding and procurement system may differ from one country to another. Thus, it seems necessary to recall that the purpose of our study was not to compare the cost of a vaccination across the different Western European countries. Estimates are in fact very different from one country to another. Such differences are primarily a reflection of a great disparity between national vaccine calendars and the funding modalities of vaccination programs which remains national prerogatives despite growing pan-EU initiatives for more convergence.

In comparison with other mass secondary prevention approaches, vaccination actually appears to be less expensive. Vaccine costs throughout life are much lower than other well-established and widely used preventive strategies in a large number of people. For instance, in France, the highest lifetime cost of vaccinating one individual would total €2,431. This cost corresponds to a women suffering from underlying conditions. This cost is still 25% less than the cost of lipid-lowering drugs or than the cost of bisphosphonates for the prevention of osteoporotic fractures. It is also half of the cost of anti-hypertensive drugs for the prevention of cardiovascular events and nearly 3 times less than the cost of DPP-4 inhibitors for the treatment of diabetes type 2 or the cost of antithrombotic drugs for the prevention of recurrent stroke (see appendix).^15^ All of these preventive medicines have been given to several millions of individuals for decades.

Our analysis has a number of limitations that should be noted. First, we only included the vaccines that were recommended in the latest official national vaccination calendar (2014 or 2015, depending on the country) and were fully or partially funded by the NHI (public funding). Some vaccines were excluded: those that are only regionally funded, as in Italy, Spain and Sweden, and those paid by third party payers (e.g., private insurers or out-of-pocket expenses). It is worth noting that this study provides a snapshot of the situation at a certain point in time. Some new vaccines may be recommended and funded by the NHI in the near future in the 7 selected European countries. Notably, this is the case for vaccination against rotavirus (as it is currently already implemented in Germany and in England), against shingles (already included in England) and against meningococcal serogroup B disease.

Second, vaccine costs are indicative of the maximal cost for one individual lifetime. They were assessed using the list prices and some conservative assumptions. Indeed, few people fully comply with all recommended vaccinations from cradle to grave; this is particularly true in adulthood. Vaccination coverage rates are disparate across different age groups, being rather good in the pediatric age group (with the exception of the MMR vaccine in some countries),^16,17^ but far from being satisfactory in adults and the elderly (except maybe in England).^18^

Third, only the fees of health professionals were taken into account in the cost of vaccine administration. Cold chain, storage, transport, logistics, disposables and any other activities related to vaccination programs, such as an awareness campaign, were not factored in.^7^ Finally, potential national rebate agreements between the vaccine manufacturers and the NHI were not considered. These agreements are usually kept confidential, making the estimation of the actual acquisition cost of vaccines difficult. Nonetheless, we ran the sensitivity analysis within a +/−30% range on the prices of vaccines to account for the uncertainty in the real acquisition cost of vaccines.

Our findings highlight the non-negligible part of vaccine administration costs. An optimization of these costs could be envisaged to make vaccination even more financially attractive. Such optimization should also promote and facilitate access to vaccinations. For instance, this could take the form of a free invitation to mass vaccination sessions (avoiding an individual visit to a medical office), systematic administration of several vaccines during the same medical visit, school vaccination programs or vaccination by health professionals other than physicians, such as nurses or pharmacists. A simplification of access to vaccinations must occur in accordance with the safety standards and the legal context of the countries concerned.

Children are specifically targeted by vaccination recommendations as they are more prone to infections. If relatively satisfactory vaccination coverage rates have been achieved in newborns and infants, much remains to be done in adulthood and the elderly in terms of appropriate vaccination coverage.^9,19-23^ Adults remain relatively unaware of the benefit of vaccinations.^24^ As the immune system wanes with age (i.e., immunosenescence), the elderly are more susceptible to infectious conditions such as influenza, pneumococcal disease or the reactivation of the varicella-zoster virus.^25-27^ Currently, a far greater number of cases of vaccine-preventable diseases may actually occur in adults, and notably seniors, who are more prone to severe and disabling diseases than younger age groups. Disease prevention is of particular importance in the elderly to diminish the risk of frailty and disability, as well as the consumption of drugs in this polymedicated population.

With the current population aging and the economic challenges faced by healthcare systems in Western Europe, vaccination policies should go beyond their focus on childhood protection and embrace a lifespan approach to vaccination, providing equal access to necessary vaccines for all age-groups. Our analysis suggests that increasing the vaccine coverage rates to a satisfactory level to achieve public health objectives would require a fairly low level of investment given the low cost of vaccination throughout life.

In this regard, our findings could be supplemented by the quantification of the health benefits provided by the national vaccination calendars at the different life stages.^28^ Future studies are thus encouraged to further streamline the resources devoted to vaccination and pinpoint the optimal vaccination calendar. Budgetary constraints, demographic changes and public health priorities are merely the main dynamics to be factored in. Ultimately, this will enhance vaccination as a smart investment providing substantial benefit that goes well beyond individual health and protects the entire population and economy against potentially troublesome and resource intensive outbreaks of infectious diseases.

In conclusion, vaccinations require a relatively low investment level per individual. Our analysis suggests that between €443 and €3,395 would be theoretically required in Western Europe to fully vaccinate one person over her/his lifetime against 10 to 17 debilitating and potentially life-threatening pathogens. Our estimates should be considered to be the maximum potential costs due to our 100% compliance assumption. Extending the vaccination scope, specifically with broader senior vaccination, where there are missed opportunities, and increasing the vaccination coverage among all age groups would bring additional public health benefits for a relatively low level of incremental investment. The conjoint search and implementation of less resource intensive administration modalities should also be encouraged to promote a more efficient administration. In our current budget-conscious era, broader and more efficient vaccination will contribute to improved public health and healthcare system sustainability.

## Materials & methods

Seven Western European countries were selected for the analysis: the 5 largest countries in terms of population, accounting for approximately 317 million (M) inhabitants in 2015 (Germany ≈81 M, France ≈64 M, Italy ≈61 M, England ≈54 M and Spain ≈46 M) and 2 additional countries from northern and southern Europe whose populations consist of ≈10 M inhabitants (Sweden and Portugal). This panel of countries constituted a good mix of public vs. private markets and regionalized vs. centralized countries. This selection ensured strong representation of the different modalities of vaccine procurement across Western Europe.

We retrieved the most recent national vaccination calendars for each country ^29-35^ (Table 1). We divided the human lifespan into 5 relevant age-groups to reflect the different vaccination stages and needs throughout life: pediatric (from birth to 24 months); pre-school (from 2 to 6 years); school (from 7 to 17 years), adult (from 18 to 64 years) and elderly (above 65 years). To determine the average number of years an individual spends in the ≥65 year age-group, we used the latest estimate of life expectancy at birth from the World Health Organization (WHO) while taking into account the difference between men and women.^36^

Within each age-group, we also made the distinction between healthy individuals and individuals suffering from one or more underlying conditions because the vaccination calendars usually recommend additional and specific vaccinations for these individuals. This is typically the case for the vaccinations against influenza, varicella, hepatitis and tuberculosis for instances.

All costs were calculated from the NHI perspective. The unit prices of each recommended vaccine were retrieved from national official sources. We used the list prices in Germany,^37^ England,^38^ France,^39^ Italy,^40^ Spain,^41^ Sweden ^42^ and Portugal ^43^ (but only for some partially funded vaccines recommended to people with underlying conditions) (Table 2). In Italy, the law imposes a minimum 50% discount rate when drugs and vaccines are sold to hospitals and local health-care units, vaccine costs were therefore adjusted to the vaccination centers (local health units) perspective to account for the impact of the law.^44^ In Portugal, we used the publicly available maximum tender price.^43^ When several brands were available for the same indication, we used the most frequently administered vaccine according to market estimates in each country.^45^ A 100% compliance rate was assumed for all recommended vaccines at any age.

**Table 2.** Levels of pricing data and administration assumptions.

**Country**	**Pricing data**	**Opportunistic vaccination**		**Co-administration**	
Germany	List price [37]	None	Cost of administration depending upon the number of vaccine valences	None	
England	List price [38]	≤6 y ≥7and ≤64 y ≥65 y	One GP visit every 2 vaccinations One GP visit every vaccination +School program for HPV vaccination One GP visit every 2 vaccinations	Birth 2 m 3 m 4 m 12 m 3 y 14 y ≥18 y ≥65 y 70 y	BCG + HB ^*^ DTacP-IPV-Hib + Pneumo cj + Rotavirus DTacP-IPV-Hib + Mgo C cj + Rotavirus DTacP-IPV-Hib + Pneumo cj Hib-Mgo C cj + Pneumo cj DTacP-IPV + MMR Td-IPV + Mgo C cj TdacP-IPV + Influenza^*^ Pneumo ps + Influenza Herpes zoster + Influenza
France	List price [39]	≤6 y ≥7 and ≤64 y ≥65 y	One pediatrician visit every 2 vaccinations One GP visit every vaccination One GP visit every 2 vaccinations	Birth ≤12 m 12 m 6 y 11–13 y 11–13 y ≥7 y ≥65 y	BCG + HB^*^ DTacP-IPV-Hib-HB + Pneumo cj Mgo C cj + MMR DTacP-IPV + Influenza^*^ TdacP-IPV + HPV HPV + Influenza^*^ Td-IPV/TdacP-IPV + Influenza^*^ Td-IPV + Influenza
Italy	List price [40,44]	None	One GP visit every vaccination	≤12 m 12 m 5–6 y ≥65 y	DTacP-IPV-Hib-HB + Pneumo cj MMR + Mgo C cj DTacP-IPV + MMR Pneumo ps + Influenza^*^
Spain	List price [41]	None	One GP visit every vaccination	2 m 4 m 6–12 m	TdacP-IPV-Hib + Pneumo cj TdacP-IPV-Hib + Pneumo cj TdacP-IPV-Hib + Pneumo cj
Sweden	List price [42]	None	One GP visit every vaccination +School program for HPV vaccination	≤12 m ≤12 m 14 y	DTacP-IPV-Hib + Pneumo cj DTacP-IPV-Hib + Pneumo cj+ HB^*^ TdaP + Influenza^*^
Portugal	List price and publicly available maximum tender price [43]	None	One GP visit every vaccination	None	

* For individuals with underlying conditions

BCG: Tuberculosis vaccine; DTacP-IPV-Hib: Diphtheria, tetanus, poliomyelitis, pertussis and hemophilus influenza type B vaccine; GP: General practitioner; HB: Hepatitis B vaccine; HPV: Human papillomavirus vaccine; Mgo C cj: Meningococcal disease C conjugate vaccine; MMR: Measles, mumps and rubella vaccine; Pneumo cj: Pneumococcal conjugate vaccine; Pneumo ps: Pneumococcal polysaccharide vaccine.

With regard to vaccine administration modalities, we took the fee for a medical appointment from official sources in Germany,^46^ England,^47^ France,^48^ Italy ^49^ and Portugal.^50^ In Spain, we used an estimate taken from a cost-effectiveness analysis of pneumococcal vaccination.^51^ In Sweden, we assumed a fee of SEK 150 per administration (and SEK 134 for the HPV vaccine) in the absence of a single fee for all age-groups and all councils. Indeed, administration fees vary greatly between and within county councils and depending on vaccines and age groups.

We also made some assumptions to better reflect local administration practices. In Germany, for instance, one medical visit per vaccine administration was assumed, but the cost of administration was dependent upon the number of vaccine valences, which was agreed upon between KV Nordrhein and Statutory Health Insurance (from €7.40 for a monovalent vaccine up to €19.50 for a hexavalent vaccine).^32^ For Italy, Spain, Sweden and Portugal, we assumed one GP appointment for every vaccination. It is important to note that Sweden has a specific school program for HPV.

For England and France, we made some additional assumptions to account for potential opportunistic vaccinations in children and the elderly during routine medical encounters. In England, we assumed one general practitioner (GP) appointment every 2 vaccinations for patients under 6-years and above 65 y of age. We assumed one GP appointment every vaccination between 7 and 64 y of age. HPV is given through specific school programs and is thus subject to specific fees. Similarly, in France, we assumed one pediatrician appointment every 2 vaccinations under the age of 6 years, one GP appointment every vaccination between 7 and 64 y of age (including HPV vaccination), and one GP appointment every 2 vaccinations above the age of 65 y.

In most countries (France, England, Spain, Sweden and Italy), the number of medical appointments for vaccine administration was also adjusted for possible co-administrations in each country. For instance, in France, the 3 doses of the DTacP-IPV-Hib-HB vaccine and the 3 doses of the pneumococcal conjugate vaccine can be co-administered at 2, 4 and 11 months in infants. Such adjustments decrease the administration cost of vaccines and better reflect current practices. Assumptions regarding the administration modalities in each country are summarized in Table 2.

As a sensitivity analysis, we varied the costs of vaccines within a +/−30% range to account for probable price variations due to competition, market type and size, as well as the vaccine life cycle. We equally varied the administration fees, but only within a +30% range, hypothesizing that medical fees are not likely to decline in the future.

## Abbreviations

*GP*: General Practitioner
*HPV*: Human Papillomavirus
*NHI*: National Health Insurance
*NVC*: National Vaccination Calendars
*WHO*: World Health Organization

## Appendix

Theoretical costs of preventive drugs in France for women.

Treatment time calculation based on French women life expectancy, among the longest one (85y).

**Table:**

Therapeutic class	Example of drugs	Treatment time	Dosing	Number of boxes	Price per box	Funding by NHI	Estimatedcost of treatment
Statin	Crestor® *rosuvastatin* 5 mg (box of 90)	25 y (60–85 y)	1/day	100	€49.64	65%	€ 3,227
Anti-hypertensive	Eupressyl®*urapidil* 60 mg (box of 30)	25 y (60–85 y)	2/day	600	€12.38	65%	€ 4,828
DPP-4 inhibitor	Januvia®*sitagliptin* 100 mg (box of 30)	20 y (65–85 y)	1/day	240	€44.00	65%	€ 6,864
Antithrombotic	Plavix®*clopidogrel* 75 mg (box of 30)	25 y (60–85 y)	1/day	300	€30.41	65%	€ 5,930
Bisphosphonate	Actonel® *risedronate* 75 mg (box of 6)	35 y (50–85 y)	1/month	70	€75.70	65%	€ 3,444

Source: ^15^ for prices per box and ^36^ for life expectancy.
